# Women’s Satisfaction with Sex Education Training: A Comparative Study Between Urban and Rural Contexts in Europe

**DOI:** 10.3390/healthcare14111463

**Published:** 2026-05-26

**Authors:** Ana Frias, Maria da Luz Barros, Ana João, Ana Galhanas, Sagrario Gómez-Cantarino, Victoria Lopezosa-Villajos, Hélia Dias, Conceição Santiago, Daniela Mecugni, Elena Castagnaro, Florbela Bia

**Affiliations:** 1 Escola Superior de Enfermagem S. João de Deus, Universidade de Évora, 7000-811 Évora, Portugal; mlb@uevora.pt (M.d.L.B.); ana.galhanas@uevora.pt (A.G.); florbela.bia@uevora.pt (F.B.); 2 Comprehensive Health Research Center (CHRC), Universidade de Évora, 7000-811 Évora, Portugal; alsjoao@hotmail.com; 3 RISE-Health, School of Health, Santarém Polytechnic University, 2005-075 Santarém, Portugal; helia.dias@essaude.ipsantarem.pt (H.D.); mconceicao.santiago@essaude.ipsantarem.pt (C.S.); 4 Department of Nursing, Health School of Santarém, 2005-075 Santarém, Portugal; 5 Faculty of Physiotherapy and Nursing, University of Castilla-La Mancha, Toledo Campus, 45071 Toledo, Spain; sagrario.gomez@uclm.es (S.G.-C.); victoria.lopezosa@alu.uclm.es (V.L.-V.); 6 Health Sciences Research Unit: Nursing (UICISA: E), Coimbra Nursing School (ESEnfC), 3004-011 Coimbra, Portugal; 7 Centre for Research in Technology and Health Services (CINTESIS), University of Porto, 4099-002 Porto, Portugal; 8 Azienda Unità Sanitaria Locale, IRCCS, 42122 Reggio Emilia, Italy; daniela.mecugni@unimore.it (D.M.); elena.castagnaro@ausl.re.it (E.C.); 9 Department of Surgery, Medicine, Dentistry and Morphological Sciences with Transplant Surgery, Oncology and Regenerative Medicine Relevance, University of Modena and Reggio Emilia, 41121 Modena, Italy; 10 Center for Interdisciplinary Research in Health (CIIS), Instituto de Ciências da Saúde, Universidade Católica Portuguesa, 1649-023 Lisbon, Portugal

**Keywords:** sexual and reproductive health, sexual education, humanisation in health, women, rural, urban

## Abstract

**Background:** Understanding participants’ perceptions of educational interventions is essential for improving the quality and acceptability of training in sexual and reproductive health. In the context of sex education, evaluating satisfaction provides relevant information about how training is experienced and perceived by participants in different social and geographical settings. **Objectives:** This study aims to explore and characterise women’s satisfaction with sex education training sessions and to analyse differences in perceived satisfaction between urban and rural contexts, focusing on organisational aspects and trainer–participant interaction. This study does not seek to evaluate the effectiveness of the intervention, but rather to examine the participants’ perceived experience of the training. **Methods:** A quantitative, exploratory, descriptive, cross-sectional study was conducted using the Women’s Satisfaction Questionnaire on Sex Education Training. The sample consisted of 180 women from different European countries, including 94 participants from urban areas and 86 from rural areas. Data were collected after the training sessions and analysed using the Statistical Package for the Social Sciences (SPSS), version 29. Descriptive and inferential statistical analyses were performed, with a significance level set at *p* < 0.05. **Results:** Overall, satisfaction levels were high, with 93.3% of participants reporting high satisfaction. The highest-rated aspects included clarity of session objectives (mean = 4.63), a supportive learning environment (mean = 4.61), and perceived relevance of the content (mean = 4.54). Satisfaction was high across both dimensions analysed—organisation of training and trainer–participant interaction. Statistically significant differences were observed according to sociodemographic variables, particularly age, education level, and area of residence, with women in rural areas reporting higher satisfaction. **Conclusions:** The findings highlight a high level of satisfaction among women participating in sex education training sessions, particularly regarding organisational quality and the interaction established with the trainer. These results provide a detailed understanding of factors associated with the participants’ perceived experience of training in different contexts. However, the study is limited to the assessment of satisfaction and does not allow conclusions to be drawn regarding the effectiveness of the training or its impact on behavioural or health outcomes.

## 1. Introduction

Sex education plays a fundamental role in promoting women’s health and well-being by providing essential knowledge about reproductive health, consent, sexuality, and healthy interpersonal relationships [1,2]. Comprehensive sexuality education also contributes to female empowerment, informed decision-making, and the prevention of sexually transmitted infections and unintended pregnancies. In this context, sexuality education represents an important public health strategy, as knowledge about biological factors and female sexual response constitutes a basis for reproductive health literacy and informed family planning [3]. Furthermore, sexuality education contributes to the achievement of the Sustainable Development Goals established in the United Nations 2030 Agenda, particularly Goal 3—Ensure healthy lives and promote well-being for all at all ages—and Goal 5—Achieve gender equality and empower all women and girls [4]. Authors such as Sanders et al. [5] highlight the need for further research to support the development of comprehensive sexuality education resources. Comprehensive sexuality education encompasses multiple dimensions of sexuality, including biological, psychological, emotional, and sociocultural factors, all of which influence sexual health and well-being throughout the life course [6,7].

In addition to the content delivered, the quality of educational experiences in sexuality education may also depend on organisational and relational aspects of training. Previous studies suggest that factors such as the learning environment, pedagogical approaches, communication style, and trainer–participant interaction can influence participants’ satisfaction and their willingness to openly discuss sensitive topics related to sexuality [8,9]. Creating supportive and non-judgmental educational spaces is therefore essential to encourage dialogue, reflection, and active participation.

Furthermore, the continuous acquisition of knowledge in this field contributes to a healthier understanding and experience of sexuality, promoting informed decision-making and sexual well-being throughout life.

Investigating differences between urban and rural populations makes it possible to identify not only the level of development, but also the specific vulnerabilities of each context [10,11]. Although rural areas have undergone changes in terms of accessibility and demographic dynamics, they still maintain traditional norms and behaviours that are socially accepted and expected. These elements, strongly rooted in local cultural values and norms, condition individuals’ behaviour and practices. Previous research suggests that important differences exist between rural and urban contexts regarding access to sexuality education, sexual health literacy, and perceptions of sexual and reproductive health. Women living in rural areas often experience reduced access to specialised health services and educational resources, greater sociocultural stigma surrounding sexuality, and fewer opportunities for open discussion about sexual health topics [12,13,14]. In contrast, urban populations generally have greater exposure to health information and services, although they may also face challenges related to information overload, cultural diversity, and variability in the quality of available information [15,16]. These contextual differences may shape participants’ expectations, engagement, and perceived relevance of sex education interventions, reinforcing the importance of adopting context-sensitive educational approaches. It is therefore crucial to understand the differences in the perception of sex education between urban and rural areas when planning care and interventions aimed at populations [17].

Factors such as socio-economic status, cultural background, and level of access to information play a significant role in the training experience and can significantly affect the effectiveness of the sex education provided.

Various authors have discussed the effectiveness of sex education in different scenarios [18,19], as well as the need to implement health policies that promote comprehensive sex education, integrating the family and the community [18].

The relevance of this study lies in the fact that it provides insight into the satisfaction of women who have taken part in sex education training in different contexts—urban and rural. The reception of such training can vary significantly between the two contexts and is influenced by aspects such as access to information, cultural norms, and socio-economic conditions, which shape the learning experience.

A number of investigations have revealed a gap in the scientific evidence in relation to comparing the experiences of women living in urban and rural areas regarding satisfaction with sex education programmes [20,21]. Although there are studies that address contextual differences in sex education, few focused specifically on women’s satisfaction in these environments. In the study by Saavedra et al. [22], a sex education programme was developed, taking into account gender, culture, and social class factors, and implemented in various regions of Portugal. Although this programme addressed issues related to sexuality, it did not focus specifically on comparing urban and rural areas or on women’s satisfaction. This highlights the need for research that specifically explores women’s experiences and levels of satisfaction with sex education training in different geographical contexts, in order to develop more effective sex education programmes tailored to their needs [22,23].

Sexual health literacy among women living in rural regions has been particularly emphasised in the literature [10,12,13]. Empowering these women to make informed decisions about their sexual and re-productive health is a fundamental step towards promoting general well-being and reducing gender inequalities in these communities. Similarly, other authors [10,20,21] confirm that rural women face unique challenges that influence sexual health literacy levels, namely inadequate access to health services, limited educational opportunities, cultural taboos about sexuality, and gender inequalities.

The results of the studies are fundamental for formulating public policies and educational practices that respond effectively to the specific needs of each group, thus revealing the particularities of each context identifying differences in sex education knowledge between groups is important because they can influence the development of future interventions and/or programmes to reduce sexual health disparities. Identifying differences in sex education knowledge between groups is important because they can influence the development of future interventions and/or programmes to reduce sexual health disparities [15].

Despite the growing recognition of the importance of sex education in promoting sexual and reproductive health, there is still limited evidence on how such training is perceived by adult women, particularly in community-based settings. Existing research has predominantly focused on knowledge acquisition, behavioural outcomes, or interventions targeting adolescents and young people, with comparatively less attention given to participants’ subjective experiences of training processes.

In this context, satisfaction constitutes a relevant indicator for assessing the perceived quality, acceptability, and adequacy of educational interventions. Exploring how women experience sex education sessions—especially in terms of organisation, content, and interaction with trainers—can provide valuable insights to inform and improve the design and delivery of future training initiatives.

Additionally, contextual factors such as place of residence may influence how training is perceived. Women living in rural and urban areas are exposed to distinct socio-cultural norms, varying levels of access to information, and different opportunities for health education, all of which may shape their expectations and experiences of sex education programmes. However, comparative evidence examining satisfaction with sex education training across these contexts remains scarce.

Therefore, this study aims to address this gap by focusing on women’s satisfaction with sex education training sessions in both urban and rural settings. This study aims to explore and characterise women’s satisfaction with sex education training sessions, and to analyse differences in perceived satisfaction between urban and rural contexts, focusing on organisational aspects and trainer–participant interaction. This study does not seek to evaluate the effectiveness of the intervention, but rather to examine the participants’ perceived experience of the training.

## 2. Materials and Methods

### 2.1. Study Design

This study adopted a quantitative, exploratory, descriptive, and cross-sectional design. It was conducted within the framework of a community-based educational intervention aimed at promoting sexual and reproductive health among women. The cross-sectional approach allowed for the assessment of participant satisfaction immediately after attending the training sessions, capturing their perceived experience at a single point in time. The exploratory and descriptive nature of the study was considered appropriate given the limited evidence available on women’s satisfaction with sex education training in different socio-cultural and geographical contexts.

### 2.2. Methodological Procedures

This study is part of the project “Educating for Sexuality: Advances in European Health” (Ed-SEX), No. 2021-1ES01-KA220-HED-000023306, which aimed to promote a holistic model of sex education within higher education and community settings. The project was implemented across different contexts, including higher education institutions (trainer and students) and community groups such as young people, women’s associations, and migrant associations.

The intervention involved the organisation of several workshops addressing topics such as sexual violence and consent, sexual diversity and emotions, functional diversity and sexuality, and sexuality and transculturality in migrant cultures.

The sessions were presented as sexuality education sessions, adopting a comprehensive and preventive approach that extended beyond the biological and clinical aspects associated with the female body. In this context, topics related to sexuality, intimate relationships, consent, contraceptive methods, prevention of sexually transmitted infections, identity, interpersonal respect, and emotional well-being were addressed. Through these sessions, the intervention aimed to promote knowledge regarding sexuality, interpersonal relationships, reproductive health, and well-being while simultaneously fostering autonomy, health literacy, and the adoption of healthy behaviours throughout the life course. The specific items included in each dimension are presented in Table 1.

The training sessions lasted approximately 90 min and were delivered to women living in both rural and urban areas in the participating countries (Portugal, Spain, and Italy).

All workshops followed a common pedagogical framework previously developed within the EdSex project, ensuring methodological consistency across countries. This framework included shared educational objectives, thematic contents, estimated duration, and participatory teaching strategies. Educational activities incorporated peer education, video viewing and discussion, poetry reading and writing, brainstorming, and reflective group discussions.

Although the facilitation approach was adapted to the participants’ characteristics, including literacy level, age group, and sociocultural context, in order to enhance engagement and comprehension, the core contents, objectives, and overall pedagogical structure of the workshops remained consistent across all participating countries.

The workshops were disseminated through community-based strategies to encourage participation. Invitations were sent in advance by email to local stakeholders, including the residents’ associations and parish councils in the areas where the sessions were conducted. Additionally, posters and informational leaflets were distributed within these institutions to further promote community engagement.

Data were collected from women who attended the sex education training sessions, resulting in a total sample of 180 participants from both urban (*n* = 94) and rural (*n* = 86) contexts. The study was conducted within the framework of the EdSex project in several European locations, namely Évora and Santarém (Portugal), Toledo (Spain), and Reggio Emilia (Italy).

The classification of participants as living in rural or urban areas was based on the self-reported place of residence provided in the sociodemographic questionnaire. The categorisation considered the administrative and territorial characteristics of the participants’ municipalities or local areas, according to the national classifications used in each participating country (Portugal, Spain, and Italy).

A non-probabilistic convenience sampling approach was used, based on voluntary participation in the training sessions. Inclusion criteria included being aged 18 years or older and providing informed consent to participate in the study.

This sampling strategy reflects the real-world, community-based nature of the intervention and intentionally enabled the inclusion of participants from diverse socio-cultural backgrounds and different countries. Such diversity allowed the study to capture a wide range of perspectives and experiences related to sex education training.

The heterogeneous composition of the sample is consistent with the exploratory aim of the study, which seeks to provide an initial and context-sensitive understanding of women’s perceived satisfaction across varied settings, rather than to generate statistically generalisable findings.

### 2.3. Data Instrument

The Satisfaction Scale [24], originally developed to assess higher education students’ perceptions of the quality of sex education training programmes, was used in this study to evaluate the participants’ perceived experience of the training sessions. Although initially designed for student populations, the scale was considered appropriate for the present study due to its focus on core dimensions of training quality, namely organisational aspects and trainer–participant interaction, which are relevant across different adult learning contexts.

Furthermore, the items included in the instrument address general aspects of educational experience such as clarity of objectives, quality of communication, learning environment, and perceived usefulness of content, which are not specific to academic settings and can be meaningfully applied to community-based training involving adult women.

All items were assessed using a five-point Likert scale, ranging from 1 (“very dissatisfied”) to 5 (“very satisfied”), with higher scores indicating greater levels of satisfaction with the training experience.

Some items in the scale were negatively worded (specifically items 4, 5, and 7 in the “teacher interaction” dimension), meaning that agreement with these statements reflected lower levels of satisfaction (e.g., lack of empathy or presence of interruptions). To ensure consistency in the interpretation of the results, these items were reverse-coded prior to analysis. Specifically, response values were recoded so that higher scores uniformly represented higher levels of satisfaction across all items (e.g., scores of 1 were recoded as 5, 2 as 4, and vice versa). This recoding was performed before calculating the dimension scores and conducting statistical analyses, thereby preventing misinterpretation and contributing to the internal consistency and reliability of the scale.

The scale was subjected to exploratory factor analysis to examine its underlying structure, resulting in the identification of two main dimensions. The first dimension, designated as “quality of the organisation of the sex education activity”, comprises items related to the physical environment, the trainer’s performance, and the content delivered. This dimension demonstrated good internal consistency, with a Cronbach’s alpha coefficient of 0.816.

The second dimension, “teacher interaction”, includes items reflecting the quality of communication and openness to dialogue between the trainer and participants. This dimension presented a Cronbach’s alpha of 0.625. Although this value is below the commonly recommended threshold of 0.70, it was considered acceptable given the exploratory nature of the study and the limited number of items included in this dimension. It is well-established that Cronbach’s alpha is sensitive to the number of items, with lower values often observed in scales with few items. Therefore, the obtained value was deemed adequate to capture the construct under analysis in this context.

The original version of the scale consisted of 19 items and showed moderate internal consistency (Cronbach’s alpha = 0.638). Following item reduction based on low item-total correlations and structural reorganisation, the final version included 9 items and demonstrated improved internal consistency (Cronbach’s alpha = 0.827), indicating good overall reliability of the instrument in this study.

### 2.4. Data Collection and Organisation

Data were collected between 30 October 2023 and 27 February 2024, after a session called ‘Conversations With Women About Women: Female Sexuality’, using the expository method and dramatisation, and lasting 45 min. After the presentation, there was a further 45 min for discussion and joint reflection. At the end of the session, all of the participants were asked to fill out the satisfaction questionnaire [25]. The questionnaire included questions related to age, country of origin, region, level of study and 14 items on satisfaction with training. Informed consent was obtained from each of the participants. Some of the participants asked for clarification of the items, and respect and autonomy were always guaranteed. In order to preserve anonymity, the questionnaires were labelled with the last three numbers of the identification document and the letter corresponding to that document.

### 2.5. Analysing Results and Statistics

Statistical analysis was performed using the Statistical Package for the Social Sciences (SPSS), version 29 (IBM Corp., Armonk, NY, USA). Prior to inferential analysis, the assumptions of normality and homogeneity of variances were assessed to justify the use of parametric tests.

Descriptive statistics were calculated for all variables, with means and standard deviations reported for continuous variables as measures of central tendency and dispersion.

Overall satisfaction scores were calculated based on the mean score obtained across the questionnaire items. For interpretative purposes, satisfaction levels were categorised as follows: mean scores between 1.00 and 2.99 were considered low satisfaction, scores between 3.00 and 3.99 indicated moderate satisfaction, and scores between 4.00 and 5.00 indicated high satisfaction.

For inferential analysis, the independent samples Student’s *t*-test was used to compare the mean differences between two groups. When comparisons involved three or more groups, one-way analysis of variance (ANOVA) was applied to detect statistically significant differences between group means. When statistically significant results were identified in the ANOVA, post hoc multiple comparisons were conducted using Tukey’s test to control for type I error.

All statistical tests were two-tailed, and the level of statistical significance was set at *p* < 0.05.

### 2.6. Ethical Aspects

The ethical principles set out in the Declaration of Helsinki were respected. The participants’ authorisation was obtained through free and informed consent, in which they expressed their willingness to participate voluntarily and to withdraw at any time if they so wished. Confidentiality was guaranteed and the data recorded, and anonymity was safeguarded.

The study was approved by the Social Research Ethics Committee of the leading university (Universidad de Castilla-La Mancha; CAU-661803-V4Z4).

## 3. Results

### 3.1. Characteristics of the Sample That Took Part in the Training

The majority of participants were over 41 years old (70.6%), while the remaining age groups were distributed as follows: 30 to 40 years old (19.4%), 21 to 29 years old (9.4%), and 18 to 20 years old (0.6%) (Table 2).

With regard to nationality, the majority of participants were from Portugal (38.3%), followed by Spain (30.6%), Italy (17.2%), and other countries (13.9%) (Table 2). As for region, 52.2 per cent of the women lived in urban areas and 47.8 per cent in rural areas (Table 1).

Regarding level of education, 46.1 per cent had completed compulsory secondary education, 18.9% had a bachelor’s degree, 20.6% had a university degree and 14.4% reported other types of training (Table 2).

### 3.2. Satisfaction with the Training

The items in which the participants showed the greatest satisfaction were as follows: ‘The teacher introduced himself and explained the theme and objective of the meeting’ (4.63), ‘The teacher maintained a relaxed attitude during the meeting’ (4.61), and ‘I believe that the topics covered have increased my knowledge in this area’ (4.54) (Table 3).

Subsequently, satisfaction with the training was assessed in the two main dimensions: quality of the organisation of the sex education activity and teacher interactions. Average satisfaction was highest in the dimension related to the ‘quality of the organisation of the sex education activity’ (M = 4.48; SD = 0.43). In the ‘teacher interactions’ dimension, the value was also very satisfactory (M = 4.46; SD = 0.60) (Table 4).

### 3.3. Satisfaction with Training Scores

The majority of participants reported a high level of satisfaction with the training (93.3 per cent), while only 6.7% showed average satisfaction (Table 5).

### 3.4. Satisfaction with Training Versus Sociodemographic Variables

Regarding age, statistically significant differences were observed in the dimension “quality of the organisation of the sex education activity” (F(2, 176) = 4.954, *p* = 0.008, η^2^ = 0.053), as well as in the “teacher interactions” dimension (Welch’s F(2, 38.904) = 4.097, *p* = 0.024, η^2^ = 0.053), indicating small effect sizes (Table 6).

Significant differences were also found in the dimension “quality of the organisation of the sex education activity” according to the country in which the training was conducted (F(2, 152) = 4.008, *p* = 0.020, η^2^ = 0.050), also reflecting a small effect size (Table 6).

With regard to region of residence, statistically significant differences were identified in both the “quality of the organisation of the sex education activity” dimension (t(178) = −2.125, *p* = 0.035, d = −0.317) and the “teacher interaction” dimension (Welch’s t(170.738) = −2.950, *p* = 0.004, d = −0.434), with small to moderate effect sizes depending on whether participants lived in urban or rural areas (Table 6).

Regarding level of education, statistically significant differences were observed in the “teacher interactions” dimension (F(2, 151) = 4.082, *p* = 0.019, η^2^ = 0.051), indicating a small effect size (Table 6).

Analysis of the a posteriori multiple comparison test with Tukey’s test, taking age into account, indicated significant differences in the ‘quality of the organisation of the sex education activity’ and ‘teacher interaction’ dimensions. People over the age of 41 had a significantly higher mean score in both dimensions than people aged between 30 and 40 (Table 6).

With regard to the country in which the training took place, Tukey’s test showed a significantly higher average value for the dimension ‘quality of the organisation of the sex education activity’ in Spain compared to Italy (4.56 vs. 4.28) (Table 6).

With regard to region of residence, it was found that women living in rural areas had a significantly higher average value for satisfaction with the ‘quality of the organisation of the sex education activity’ and satisfaction with the ‘interaction of the teacher’ than women living in urban areas (Table 6).

Tukey’s test showed statistically significant differences in the ‘teacher interaction’ dimension in terms of level of education. Thus, participants with an compulsory secondary education degree had a higher average value for satisfaction with the trainer than participants with a bachelor’s degree (4.57 vs. 4.23) (Table 6).

## 4. Discussion

In this study, the training sessions were characterised by an informal and participatory approach, which appears to have contributed substantially to the high levels of satisfaction reported by the participants. These findings are consistent with previous research [26], suggesting that participatory methodologies promote more open, reflective, and meaningful engagement with topics related to sexuality, including perceptions of the body, desires, and intimate relationships.

A distinctive feature of this study is the heterogeneity of the sample, which included women from different countries, socio-cultural backgrounds, and geographical contexts. Rather than representing solely a limitation, this diversity reflects the real-world conditions in which community-based sex education interventions are commonly implemented. From an exploratory perspective, this multicultural composition constitutes an important strength, as it enabled the inclusion of a broad range of experiences and perceptions. Consequently, the study provides a more nuanced and context-sensitive understanding of how sex education training is perceived across different settings. Such variability is particularly relevant in the field of sexual and reproductive health, where cultural, social, and contextual factors strongly influence individual experiences and interpretations.

The informal and dialogical nature of the training sessions appears to have played a central role in shaping participant satisfaction, suggesting that less rigidly structured educational environments may foster greater engagement, perceived relevance, and empowerment. Beyond immediate satisfaction, participatory approaches may also contribute to longer-term transformations in women’s relationships with their bodies and sexuality. This interpretation is supported by Edwards [26], who highlights that reflective and participatory strategies in sexuality education can promote more positive self-perceptions, enhance understanding of personal desires and experiences, and potentially improve intimate relationships and overall well-being.

The higher satisfaction levels observed among participants over 41 years old may also be associated with the participatory and reflective methodologies adopted during the training sessions, including peer discussion, videos, reflective activities, and collective sharing of experiences. These approaches appeared to facilitate open communication and create a supportive environment in which the participants felt comfortable discussing sensitive topics related to sexuality. Although the present study did not specifically evaluate which pedagogical strategies were preferred by each age group, informal observations during the sessions suggested that older women particularly valued opportunities for dialogue, mutual listening, and experiential reflection. Future studies should further explore age-related preferences regarding educational methodologies in sexuality education contexts.

An especially noteworthy finding concerns the significantly higher levels of satisfaction reported by women living in rural areas across both the “quality of the organisation of the sex education activity” and “interaction with the trainer” dimensions. Although this finding may initially appear counterintuitive, given the structural disadvantages often associated with rural settings, it may be interpreted through a socio-cultural and access-oriented perspective. In this context, higher satisfaction levels may reflect previously unmet educational needs, as well as the value attributed to safe, participatory, and supportive learning opportunities.

As highlighted by Chávez-Somoza and Vilchez-Salés [13], rural populations frequently experience limited access to sexual health information and services, which is associated with increased vulnerability to adverse outcomes such as unplanned pregnancies, sexually transmitted infections, and sexual violence. Within this framework, the training intervention may have been perceived not only as informative but also as a rare and valued opportunity for dialogue, thereby amplifying the satisfaction levels.

These findings may also be interpreted in light of the persistent structural inequalities affecting access to health and sexual education services in rural regions of Southern Europe. In Portugal, Spain, and Italy, rural territories continue to experience shortages of specialised healthcare professionals, limited availability of sexual and reproductive health services, ageing populations, and geographical barriers that restrict access to continuous health education initiatives [12,14,16,27]. In Spain, rural areas associated with the phenomenon of “*España vaciada*” frequently require long travel distances to access primary healthcare and specialised services, contributing to territorial inequities in healthcare access, according to Alloza et al. [27]. Similar challenges have been described in sparsely populated regions of Portugal and Italy, where healthcare provision is constrained by demographic decline, workforce shortages, and service centralisation [12,14,27]. Within this context, the sex education sessions developed in the present study may have represented one of the few opportunities for women living in rural communities to engage in participatory, supportive, and culturally sensitive discussions about sexuality and reproductive health. Consequently, higher satisfaction levels among rural participants may reflect not only the quality of the intervention itself, but also the perceived value of accessing educational spaces that respond to previously unmet informational and relational needs, as also suggested by previous studies [14,19,27].

Moreover, the role of socio-cultural stigma in shaping these experiences should not be underestimated. In many rural settings, entrenched norms surrounding sexuality may constrain open discussion, reinforcing silence and limiting opportunities for critical engagement [13]. In this sense, the training sessions may have functioned as “safe spaces” that temporarily disrupted these normative constraints, enabling the participants to express concerns and explore sensitive topics in a supportive environment. This may partially explain the higher satisfaction reported, not necessarily as an indicator of superior intervention quality, but as a reflection of previously unmet needs.

These findings also invite critical reflection on prevailing assumptions within sexuality education. The tendency among educators to conceptualise rural communities as uniformly conservative and characterised by low sexual health literacy [16] risks reproducing reductive narratives that may not fully capture the heterogeneity of these populations. Furthermore, as noted by Ouahid et al. [28], stigma associated with seeking sexual health information can further exacerbate informational inequalities, reinforcing barriers to access. In contrast, women in urban contexts may be exposed to a multiplicity of information sources, including digital media, which, while increasing access, may also contribute to information overload and variable content quality [15]. This paradox highlights that a greater availability of information does not necessarily translate into higher satisfaction or more meaningful learning experiences.

The limited availability of specialised educators, particularly in rural areas, remains a structural constraint that may compromise the quality and sustainability of sexuality education initiatives. At the same time, the assumption of homogeneity within these communities may lead to the implementation of standardised programmes that fail to respond to local needs and lived experiences [13,16]. This underscores the importance of adopting context-sensitive approaches that move beyond deficit-based perspectives and engage with the specific socio-cultural dynamics of each setting.

In urban environments, the diversity and complexity of populations require pedagogical approaches that are not only inclusive but also adaptable and responsive. Participatory models that prioritise dialogue, reflexivity, and co-construction of knowledge appear particularly well-suited to these contexts [16]. Additionally, the integration of digital technologies, including telehealth, offers promising avenues for expanding access to sexuality education, although their effectiveness remains contingent on issues of digital literacy and equity [14].

Across both contexts, satisfaction emerges as a key—yet conceptually limited—indicator of training quality. While high levels of satisfaction, as observed in this study, are associated with increased motivation, engagement, and perceived usefulness, they do not necessarily reflect deeper learning processes or behavioural change. In this regard, the strong performance observed in the dimensions “quality of organisation of sexual education activity” and “teacher interactions” points to the centrality of pedagogical design and relational dynamics in shaping participants’ experiences. In particular, the ability of trainers to structure content in a coherent, relevant, and accessible manner, while fostering a supportive and non-judgmental environment, appears to be a critical determinant of perceived quality.

From a theoretical perspective, these findings align with constructivist and critical pedagogical approaches, which conceptualise learning as an active, situated, and relational process. As argued by Jones [16], women’s sexual education should be understood not merely as the transmission of information, but as a process of knowledge construction that promotes agency and supports individuals in making informed decisions about health, illness, and care. In this sense, the emphasis on participant engagement, dialogue, and experiential learning observed in the present study may be seen as key mechanisms underpinning both satisfaction and the broader educational value of the intervention.

In this context, the organisation of the content covered particularly emphasised the importance of screenings, as an essential component in promoting health and preventing disease. Raising awareness about the need for cervical cancer screening tests and the importance of regular medical check-ups allows for the early detection of pathologies [29]. Promoting the early detection of these conditions can save lives, especially when supported by sexuality education that informs and empowers women.

The training sessions were designed to ensure a safe and welcoming environment that would facilitate the presentation of content and joint reflection on topics considered sensitive.

Teacher interactions in the context of sexual education play a central role in the effectiveness of the teaching–learning process, acting as a mediating element between the content transmitted and the experiences of the participants. The analysis of teaching narratives has highlighted the importance of adopting pedagogical practices that respect the differences and prior knowledge of the participants [30]. These interactions, when based on a dialogical and judgment-free model, promote the development of personal and social skills, encouraging informed and responsible attitudes towards sexuality.

The findings are consistent with those of Zannata et al. [31], who point out that, depending on the context, the success of training is linked to a flexible pedagogical stance, based on dialogue and the rejection of authoritarianism, prejudice, and a one-sided view. Adequate training for professionals is therefore essential to guarantee effective and respectful educational practices. Several authors [32] emphasise the importance of having well-prepared professionals, not only in technical terms, but also with pedagogical skills that integrate and value the diversity of learners. Other researchers emphasise the need for up-to-date training practices, which take into account social, digital, cultural, political and economic factors in line with the concrete reality of the population [30]. In this sense, the quality of the pedagogical relationship established by the trainer contributes directly to levels of satisfaction with the training.

The shortage of professionals and services specialising in sexual health remains an obstacle in rural regions. The main barriers identified include low supply, high costs, and fragmented health systems [12,14]. Demand for these services is also conditioned by low levels of awareness and economic obstacles [14].

In urban regions, despite greater access to services, there is an information overload that makes it difficult to distinguish between reliable and unreliable content. The social and cultural diversity of urban populations poses an additional challenge to the application of uniform sex education models [16]. These differences require educational strategies adapted to different identities and experiences. Programmes must be inclusive and participatory, integrating various gender identities and sexual orientations, so that they can respond effectively to the diverse needs of the population [33,34].

During the study, we were aware that we were intervening in a space of experience and dynamics between women, around a sexuality marked by silences, controversies, and historical omissions in this area. Aggleton and her collaborators [35] emphasise that the contemporary context reveals a growing trend of conservatism, homophobia, transphobia, and ideological conflicts around gender issues, underpinned by patriarchal norms, colonial legacies, and neoliberal agendas. Other authors refer to the existence of socio-cultural stigma and the scarcity of specialised services and educators trained in the area of sexuality [12,13,14,36].

Despite these difficulties, there is a record of professionals committed to providing quality sexual and reproductive education [37], which is considered essential to ensure general well-being and health [38]. These findings highlight the need to implement effective, culturally-adapted programmes and involve families, communities, and local authorities [13]. It is essential to create safe spaces for expression and to consider the social dynamics that shape women’s decisions [14]. Social dynamics are particularly relevant, as they influence the way the participants interact with each other and with the trainer, attitudes towards the topics covered (e.g., gender, sexuality, cultural values), the level of openness or resistance to sharing and discussion, and the impact of peer pressure and social norms on individual behaviour.

Community involvement is essential in both contexts [10]. The development of this educational intervention involved liaising with health and education organisations and local authority representatives. This inter-institutional collaboration was decisive in guaranteeing the adherence and impact of the actions developed. In rural areas, the participation of local authorities facilitates acceptance of the intervention [13], while in urban areas, it is essential to consider the heterogeneity and complexity of communities [16].

Public policies must strengthen health systems. In rural areas, it is essential to expand the supply of services and training activities; in urban areas, it is necessary to combat misinformation and adopt intersectional approaches [12,14,16].

In light of the results obtained, it is recommended that new studies be carried out on pedagogical interventions in this area, particularly with regard to satisfaction with sex education training. Such studies should seek a holistic approach, centred on empowering women to make informed and conscious decisions about their lives and their sexual and reproductive health.

## 5. Limitations/Strengths

This study has several limitations that should be considered when interpreting the findings. First, a non-probabilistic convenience sampling strategy was used, which may limit the representativeness of the sample and restrict the generalisability of the results. Although the sample size (*n* = 180) was adequate for exploratory analyses, participants were self-selected volunteers who may have been more interested, receptive, or comfortable discussing sexuality-related topics than the general population. As participation involved attending a 90 min training session on sexual and reproductive health, this predisposition may have contributed to higher levels of engagement and satisfaction, potentially inflating the overall satisfaction scores reported.

The heterogeneity of the sample also constitutes a relevant limitation. Participants differed in terms of age, socio-cultural background, and country of residence (Portugal, Spain, and Italy), factors that may have influenced perceptions of and satisfaction with the training sessions. Although this diversity reflects the real-world context of community-based interventions, it may have reduced the comparability of findings across subgroups and limited the identification of more homogeneous patterns.

Another limitation concerns the assessment instrument. Although the questionnaire demonstrated satisfactory internal consistency in the present sample, it was originally developed for university students and was not specifically validated for adult women in community-based sex education contexts. Therefore, issues related to construct validity and contextual equivalence should be interpreted with caution.

Furthermore, the study focused exclusively on satisfaction as an outcome measure. While satisfaction is an important indicator of perceived quality and acceptability, it does not allow for conclusions regarding the effectiveness of the intervention or its impact on the participants’ knowledge, attitudes, or behaviours. Similarly, the questionnaire did not directly assess the specific contribution of pedagogical approaches or participatory methodologies used during the sessions, such as peer education, videos, poems, or brainstorming activities. Consequently, interpretations regarding the influence of these approaches should be considered exploratory and contextual rather than causal conclusions directly supported by the data.

Future research should consider the use of more homogeneous or stratified samples, particularly regarding age, socio-cultural background, and geographical context, in order to enable more robust comparisons. Longitudinal or quasi-experimental designs would also contribute to a more comprehensive evaluation of sex education interventions, including their potential impact beyond the participants’ perceived experience and immediate satisfaction.

Despite these limitations, this study contributes to the existing literature by providing evidence on women’s satisfaction with sex education training in community-based and multicultural contexts. Research focusing on adult women’s perspectives in this field remains limited, particularly regarding comparisons between rural and urban settings and across different European regions. Therefore, this study offers relevant descriptive insights into factors associated with the perceived quality and acceptability of sex education interventions.

## 6. Implications for Practice

The findings of this study contribute to a better understanding of the factors associated with women’s satisfaction with sex education training, particularly in relation to session content, the learning environment, and trainer–participant interaction. These aspects appear to play a relevant role in shaping the participants’ perceived experience of training, both in urban and rural contexts.

From a practical perspective, the results may inform the design and organisation of sex education training programmes by highlighting elements valued by the participants, such as clear communication, supportive learning environments, and the perceived relevance of the topics addressed. Attention to these factors may contribute to improving the perceived quality and acceptability of training across different settings.

The differences observed between rural and urban participants suggest that contextual factors should be considered when planning and delivering educational interventions. Women in rural areas may face barriers related to access to services, socio-cultural stigma, and the limited availability of specialised resources, whereas urban contexts may present challenges related to information overload and population diversity [14,16]. Tailoring training approaches to these specific contexts may enhance participant engagement and satisfaction.

However, these implications should be interpreted with caution, as the present study focused exclusively on satisfaction and did not assess the effectiveness of the training or its impact on behavioural or health outcomes.

Overall, this study highlights the importance of adopting context-sensitive and participant-centred approaches in sex education, emphasising the role of pedagogical organisation and interpersonal dynamics in shaping the training experience, as supported by previous literature [14,16].

## 7. Conclusions

The findings of this study indicate high levels of satisfaction among women participating in sex education training sessions. Satisfaction was analysed across two main dimensions: the quality of the organisation of the training and the interaction between the trainer and participants, both of which were positively evaluated.

The results suggest that elements such as the relevance of the content, the pedagogical approach adopted, the learning environment, and the quality of trainer–participant interaction are important factors associated with the participants’ perceived experience of training. In particular, the use of interactive and participatory methods, including discussion-based activities and role-playing, was positively valued by participants and contributed to favourable perceptions of the training process.

The findings also highlight the importance of considering the socio-cultural context in which training takes place, as differences observed between groups suggest that participants’ experiences may vary according to contextual and demographic factors. These aspects reinforce the need for context-sensitive and participant-centred approaches in the planning and delivery of sex education training.

This study provides a descriptive analysis of women’s satisfaction with sex education training and contributes to the understanding of factors associated with the perceived quality of such interventions. However, the results should be interpreted with caution, as the study did not assess the effectiveness of the training or its impact on knowledge, attitudes, or behaviours.

In summary, well-organised training sessions that promote clear communication, supportive learning environments, and meaningful interaction between trainers and participants are associated with higher levels of satisfaction and a more positive training experience.

## Figures and Tables

**Table 1:** Specific items included in each dimension.

Dimension	Specific Items Included
Sexual education	Concept of sexuality; affective relationships; consent; interpersonal communication; diversity; sexual and reproductive rights
Reproductive health	Contraceptive methods; menstrual cycle; fertility; family planning
Infection prevention	Sexually transmitted infections (STIs); modes of transmission; condom use; prevention strategies; screening
Women’s health	Hormonal changes; gynaecological health; self-care; preventive examinations; physical and emotional well-being
Relationships and behaviour	Mutual respect; decision-making; peer pressure; personal boundaries; emotional responsibility
Well-being and self-esteem	Body image; self-esteem; emotional regulation; confidence; promotion of healthy habits

**Table 2:** Sociodemographic characterisation (*n* = 180).

Variables	Dimensions	*n*	%
Age	18–20	1	0.6
	21–29	17	9.4
	30–40	35	19.4
	>41	127	70.6
Country of origin	Spain	55	30.6
	Italy	31	17.2
	Portugal	69	38.3
	Other	25	13.9
Region	Urban	94	52.2
	Rural	86	47.8
Level of studies	Compulsory secondary education	83	46.1
	Bachelor’s degree	34	18.9
	University	37	20.6
	Others	26	14.4

**Table 3:** Minimum, maximum, mean, and standard deviation values for satisfaction with training items.

Item Satisfaction	Minimum	Maximum	Mean	Standard Deviation
1. The space used for the training was adequate.	1	5	4.51	0.705
2. The lighting and temperature of the room were pleasant.	2	5	4.40	0.674
3. You found the furniture comfortable to use.	1	5	4.29	0.870
4. The interview scenario took place in an unfavourable space.	1	5	2.53	1.435
5. There were many external interruptions during the interview.	1	5	2.21	1.349
1. The teacher introduced himself and explained the theme and objective of the meeting.	3	5	4.63	0.589
2. The teacher maintained a relaxed posture during the meeting.	3	5	4.61	0.573
3. Believes that the non-verbal language used by the teacher was in consonance with the verbal language.	1	5	4.46	0.772
4. During the training, did you feel listened to by the trainer?	1	5	4.49	0.758
5. Did you perceive movements or sounds in the teacher that would indicate discrepancy?	1	5	2.14	1.319
6. Do you consider that the language used was respectful and understandable?	1	5	4.46	0.929
7. Did you feel little empathy and/or acceptance from the teacher during the dialogue?	1	5	2.53	1.594
8. Do you consider that the expert and the training provided were within the timeframe?	1	5	4.42	0.825
9. Has the teacher enabled you to develop your views with reference to the questions asked?	1	5	4.44	0.785
10. In general, are you satisfied with the development of the training?	2	5	4.57	0.686
1. I feel that the topics covered have increased my knowledge in this field.	2	5	4.54	0.696
2. I believe that topics have been addressed that can be applied in day-to-day life in their future profession.	1	5	4.17	0.996
3. I think that the approach to the subject was repetitive.	1	5	2.39	1.447
4. In my opinion, a more in-depth approach to the topics covered would be necessary.	1	5	3.33	1.495

**Table 4:** Dimensions of satisfaction with training.

Satisfaction Dimensions	Minimum	Maximum	Mean	Standard Deviation
Quality of the organisation of the sex education activity	3.17	5.00	4.4778	0.43096
Teacher interactions	2.33	5.00	4.4630	0.60249

**Table 5:** Training satisfaction scores.

Satisfaction with Training Scores	*n*	%
Medium Satisfaction	12	6.7
High Satisfaction	168	93.3
Total	180	100.0

**Table 6:** Significance of the differences in the dimensions of satisfaction with sex education training according to sociodemographic variables.

Variables	Categories	Quality of Organisation M (SD)	Statistic	*p*	Effect Size	Teacher Interaction M (SD)	Statistic	*p*	Effect Size
Age	21–29	4.33 (0.33)	F(2, 176) = 4.954 ANOVA	0.008 **	η^2^ = 0.053	4.33 (0.44)	F(2, 38.904) = 4.097 Welch	0.024 *	η^2^ = 0.053
	30–40	4.32 (0.39)				4.22 (0.75)			
	>41	4.54 (0.44)				4.55 (0.55)			
Country of origin	Spain	4.56 (0.44)	F(2, 152) = 4.008 ANOVA	0.020 *	η^2^ = 0.050	4.42 (0.63)	F(2, 152) = 2.274 ANOVA	0.106	η^2^ = 0.029
	Italy	4.28 (0.45)				4.28 (0.64)			
	Portugal	4.48 (0.43)				4.56 (0.58)			
Region	Urban	4.41 (0.45)	t(178) = −2.125 Independent Samples *t*-Test	0.035	d = −0.317	4.34 (0.67)	t(170.738) = −2.950 Welch’s *t*-test	0.004	d = −0.434
	Rural	4.55 (0.40)				4.60 (0.49)			
Level of education	Compulsory secondary education	4.54 (0.43)	F(2, 151) = 3.212 ANOVA	0.047	η^2^ = 0.041	4.57 (0.62)	F(2, 151) = 4.082 ANOVA	0.019	η^2^ = 0.051
	Bachelor’s degree	4.37 (0.44)				4.23 (0.54)			
	University	4.36 (0.42)				4.38 (0.64)			

Note: Values are presented as mean (standard deviation). Group comparisons were performed using one-way analysis of variance (ANOVA) or independent samples *t*-tests, according to the number of groups analysed. Welch corrections were applied when the assumption of homogeneity of variances was violated. Effect sizes are reported as eta squared (η^2^) for ANOVA analyses and Cohen’s d for *t*-tests. Statistical significance was set at *p* < 0.05. * *p* < 0.05; ** *p* < 0.01.

## Data Availability

The raw data supporting the conclusions of this article will be made available by the authors on request.
